# MYH9: Structure, functions, and therapeutic implications in cancer and genetic disorders

**DOI:** 10.1016/j.gendis.2025.101977

**Published:** 2025-12-11

**Authors:** Shayan Emami, Amirreza Mazloomi, Fatemeh Ziadloo, Shaghayegh Hosseinzadeh, Hassan Saeedi, Azin Khoshghiafeh, Mohammad Reza Ahmadifard

**Affiliations:** a Department of Medical Genetics, School of Medicine, Babol University of Medical Sciences, Babol 4774547176, Iran; b Cellular and Molecular Biology Research Center, Health Research Institute, Babol University of Medical Sciences, Babol 4774547176, Iran

**Keywords:** MYH9, MYH9-RD, ncRNA, Neoplasms, NMIIA, Oncogene, Therapeutic target

## Abstract

The myosin heavy chain 9 (MYH9) gene encodes non-muscle myosin heavy chain IIA (NMIIA), a vital protein involved in fundamental cellular activities, including movement, cell division, and signal transmission. Mutations in MYH9 were initially linked to autosomal dominant disorders collectively termed MYH9-related diseases (MYH9-RD). In recent years, MYH9 has gained significant attention for its pivotal roles in various cancers. However, despite extensive research, its exact contributions to cancer progression remain incompletely understood. Targeting MYH9 through approaches such as non-coding RNAs, small molecules, or gene therapy presents a promising avenue for advancing cancer treatment. Additionally, the dual role of MYH9 in MYH9-RD and cancer raises the intriguing question: are individuals with MYH9 mutations predisposed to or protected from cancer? This review aims to present the structure, functional significance, and clinical associations of MYH9, with an emphasis on its contributions to MYH9-RD and cancer progression. Furthermore, it examines MYH9’s regulatory interactions with non-coding RNAs and its potential applications as a therapeutic target, offering insights into strategies such as RNA interference and CRISPR-based gene editing for cancer treatment.

## Introduction

Cancer ranks as the primary cause of global mortality across diverse populations and regions. In 2022, cancer accounted for nearly 20 million new diagnoses and resulted in approximately 9.7 million deaths. About 20% of people will eventually get cancer in their lifetime, and one in nine men and one in twelve women die from it.^1^ Recent studies indicate that MYH9 plays an important role in cancer diagnosis, progression, and treatment, other than its original role, which was a mutation in the gene itself, which led to autosomal dominant disorders, alternatively referred to as myosin heavy chain 9 (MYH9)-related diseases (MYH9-RD). The Myosin superfamily consists of more than 30 classes of proteins. There are three non-muscle myosin II heavy chain isoforms and including non-muscle myosin heavy chain IIA (NMHCIIA), NMHCIIB, and NMHCIIC, which are being discussed herein in this review.^1^, ^2^, ^3^ The MYH9 gene on chromosome 22 codes for NMIIA. MYH9 is capable of doing multiple cellular tasks, including interacting with actin and utilizing magnesium-dependent ATP hydrolysis to generate mechanical force, and is classified as an actin molecular motor.^4^ This mechanical force facilitates cellular motility and division, intracellular molecular transport, preservation of cell polarity during cytokinesis, and signaling.^5^

The role of the MYH9 gene varies across different tumor types, functioning either as a tumor promoter or suppressor. Notably, more than 90% of fatalities in patients with malignant tumors are ascribed to metastasis, a multifaceted process involving tumor cell migration and invasion. A pivotal aspect of metastasis is the alteration in tumor cell adhesion, enabling cells to detach from the primary tumor, disseminate to distant organs, and subsequently establish secondary metastatic sites.^6^ Structural proteins are essential in this process, as they facilitate dynamic cytoskeletal remodeling and supply the required energy for cell migration. These mechanisms are often associated with poor clinical outcomes.^7^

Furthermore, MYH9 is associated with the emergence of drug resistance in multiple tumors.^8^ Some therapeutic approaches have been devised to counter this resistance; further investigations are needed to evaluate their broader applicability and identify more effective alternatives. The underlying mechanisms driving drug resistance remain a significant area of research focus.^9^

Promising therapeutic strategies targeting MYH9 include Cinobufotalin (CB), Enkurin (ENKUR), saponin monomer 13 (DT-13), and immunotherapy.^10^, ^11^, ^12^ In addition, certain microRNAs (miRNAs) and aminated fullerene have demonstrated potential efficacy in specific malignancies.^13^ Despite these advancements, continued research is essential to refine these approaches and enhance their clinical relevance.

This review aims to present the significance of structure and function, its association with MYH9-RD, its role in cancer progression, the regulatory interplay with non-coding RNAs (ncRNAs), and the potential for therapeutic drugs. The investigation of MYH9 stands poised to offer critical insights into cancer biology and cancer medicine; understanding the underlying genetic and molecular pathways, including those involving MYH9, has never been more pivotal. Promising therapeutic interventions that target MYH9 or its regulatory interactions with ncRNAs may open a new era in the fight against cancer to achieve precision and individualization in treatment modalities.

## Molecular structure and function

### Molecular structure of MYH9

MYH9 is a gene consisting of roughly 107 kbp, containing 41 exons in total, located at chromosome locus 22q12.3. The open reading frame beginning with the second and ending with the 41st exon does not contain any translation, but rather codes for a polymorphic protein, NMHCIIA of chain length 1960 amino acids in size.^14^ Specifically, the analysis of the basal promoter region of MYH9 showed typical characteristics of a housekeeping gene represented by a high content of GC boxes, large GC content, and a total lack of a TATA box.^15^ Further, two enhancer regions were mapped within intron 1, 23–150 kb downstream of the promoter.^4^ The three myosin heavy chains with molecular weights of 230 kDa each, encoded by these genes, that are MYH9, MYH10, and MYH14, are distinct myosins. In contrast, the MYL6, MYL9, and MYL12 genes encode a family of light chains, each ranging from 17 to 20 kDa. The multimeric protein product of these genes is referred to as NMII.^16^

NMIIA is a hexametric assembly that consists of two essential light chains (17 kDa) that assist in maintaining the structure of the heavy chain, two regulatory light chains (20 kDa) that modulate the act of myosin, and a heavy chain dimer (230 kDa).^9^ When individual hexamers combine, myosin minifilaments are formed; these bipolar filaments are made up of up to 30 nm hexamers and typically measure 300 nm. These minifilaments, therefore, possess ATP-dependent motor activity and can cause tension between antiparallel actin filaments.^17^

**Figure 1:**
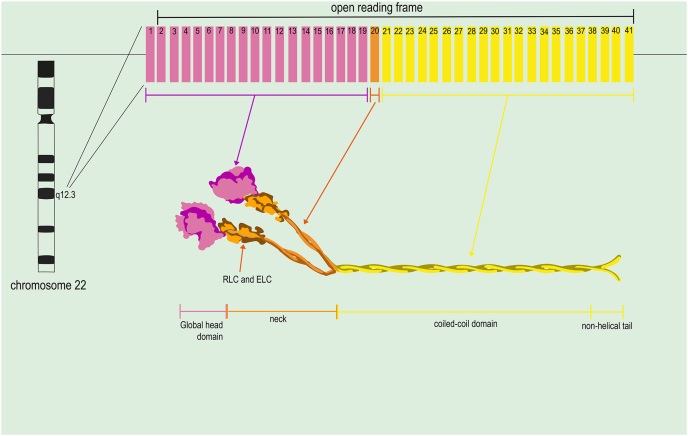
Organization of the MYH9 gene and protein. The genomic structure of the MYH9 gene is characterized by its specific arrangement of exons and introns, which play a crucial role in its function and regulation. MYH9 extends over 107 kbp on chromosome 22q12.3. The gene includes 41 exons, with the open reading frame spanning from exon 2 to exon 41. The gene encodes non-muscle myosin heavy chain IIA, a protein consisting of 1960 amino acids.

The heavy chain of NMIIA can be divided into two parts: the N terminus and the C terminus.^18^ The N terminus, the functioning motor, is the motor head domain that facilitates actin binding and force generation through MgATP utilization (Mg^2+^ being an essential cofactor). Exons 2–19 are responsible for coding this domain 9. The myosin light chain exon 20 encodes the neck (ELC and RLC) binding is encoded by exons 19 and 20; this is called the “neck” or lever arm, which moves to turn the force produced by the motor domain into movement.^19^ Within the neck region of NMIIA, glutamine-rich (IQ) motifs bind to calmodulin or calmodulin-like light chains, though their length can vary considerably. This segment functions as a mechanical lever arm, converting subtle structural shifts in the catalytic domain into precise, nanoscale displacements at the distal end of the lever.^20^ The coiled-coil domain of NMIIA is encoded by exons 21 through 40, while the tail region, or C-terminal domain, contributes significantly to helical homodimer formation, a feature shared with other myosin family members (Fig. 1).^21^ The terminal 34 amino acids, which form a non-helical tail, are encoded by alternatively spliced exon 41; this segment exhibits sequence variation across different isoforms.^9^

The myosin motor domain is essentially made up of four subdomains interconnected by flexible linkers. The amino-terminal subdomain contains an SH3-like motif typical of the myosin II class molecules. This particular motif is not found in other classes of myosin ^22^. RLC (regulatory light chain) controls chiefly the activation and deactivation of NMIIA by way of the phosphorylation or dephosphorylation of Ser, Tyr, and Thr residues.^9^ In addition to this, the ATP hydrolytic activity in NMII proteins is regulated mainly by controlling the reversible phosphorylation of some conserved residues in the RLCs, most notably with respect to the necessary serine 19 and the tubulin-enhancing serine 18.^23^ The RLCs are phosphorylated by many kinases, which include Rho-associated coiled coil-containing kinase (ROCK) activated by the small GTPase RhoA.^4^

The 60%–80% identity in the amino acid sequences of heavy chains between NMIIs and smooth muscle myosin results in comparable properties, encompassing the capacity of individual molecules to shift into a folded conformation that can align into small bipolar filaments following activation by RMLC phosphorylation.^16^

The mouse ortholog of MYH9 extends over 81 kbp, exhibits identical genomic organization to the human gene, encodes a protein of comparable length, and demonstrates 97.1% amino acid similarity with the MYH9 protein. This suggests that the mouse serves as an effective model for investigating the role of MYH9 in eukaryotic cells.^24^ From fungi to mammals, the MYH9 gene is an example of a highly conserved gene.^4^

### Cellular function

The MYH9 gene product is involved in cellular adhesion,^25^ polarity, migration, division, and signaling.^26^, ^27^, ^28^ NMIIA is an actin-based motor protein that forms bipolar filaments due to electrostatic interactions at the C-terminal ends of the heavy chains. NMIIA can also play a role in development. For example, it was shown to play a role in mouse embryonic development, and NMIIA knockdown reduced visceral endoderm development.^29^ NMIIA can play different roles in regulating signaling activities in cells. These roles can be achieved by interacting with signaling pathways. NMIIA is a structural component of the myosin-actin system and can interact with various proteins involved in signaling pathways. These interactions can help regulate signaling activities. For example, they can be involved in PI3K/AKT, mTOR, and Wnt/β-catenin signaling pathways.^30^^,^^31^

### MYH9-related diseases

#### Overview of MYH9-RD

MYH9-RD is termed a collection of autosomal dominant disorders that arise from mutations in the MYH9 gene in humans.^32^ A primary characteristic of MYH9-RD is congenital thrombocytopenia, which is often accompanied by macrocytosis of platelets and inclusions of the NMHCII-A protein in neutrophil cytoplasm.^33^ For several individuals, these hematological symptoms are the only signs of the disease that they experience throughout their whole life. Yet, the majority of patients with MYH9-RD will eventually develop further late-onset symptoms, including sensorineural deafness, kidney problems, presenile cataracts, and raised liver enzymes throughout the course of their condition.^34^

Historically, MYH9-RD was divided into five syndromic conditions previously considered separate disorders: May-Hegglin anomaly (MIM155100), Sebastian syndrome (MIM605249), Fechtner syndrome (MIM 153640), Epstein syndrome (MIM 153640), and autosomal dominant deafness DFNA17 (MIM 603622) (Table 1).^35^ After identifying the MYH9 gene as the common cause, these conditions were unified under the MYH9-RD diagnosis. MYH9-RD represents the most common type of inherited thrombocytopenia; however, it remains rare, with an estimated prevalence of 3 per 1,000,000, according to data from an Italian registry.^36^ The actual prevalence may be higher due to mild, often misdiagnosed cases. Global reports of MYH9-RD have been made, and there is no indication that the prevalence varies by ethnicity.

**Table 1:** Comparison between MYH9-RD syndromes: Overview of key features of major disorders, including common mutations, hematological and extra-hematological findings, as well as clinical severity.

Syndrome	Common mutations	Hematological features	Extra-hematological features	Notes
May-Hegglin	R702 and head domain	Severe thrombocytopenia, giant platelets, Döhle-like inclusions	Rare kidney or hearing issues, mild bleeding	Considered mildest MYH9-RD
Sebastian	Similar to May-Hegglin	Moderate thrombocytopenia, giant platelets, Döhle-like inclusions	Occasional hearing loss or cataracts	Similar to May-Hegglin with variable extra-hematological findings
Fechtner	D1424 and the coiled-coil domain	Severe thrombocytopenia, giant platelets, Döhle-like inclusions	Sensorineural hearing loss, kidney disease, cataracts	Notable for multi-system involvement
Epstein	R1165, D1424	Severe thrombocytopenia, giant platelets, Döhle-like inclusions	Progressive hearing loss, aggressive kidney disease, occasional cataracts	One of the most severe MYH9-RD types

The severity of thrombocytopenia among MYH9-RD patients varies widely, ranging from mild reductions in platelet count to severe cases. Platelet counts are generally stable over time, and platelet function remains normal. Bleeding severity corresponds to platelet counts, with most patients experiencing minimal bleeding, often limited to skin bruising. Significant hemorrhages occur primarily following hemostatic challenges, such as surgery or childbirth.^37^ About 28% of patients experience spontaneous mucosal bleeding, including menorrhagia, nosebleeds, and gum bleeding, though life-threatening bleeding events are uncommon.

Extreme platelet macrocytosis, which is an essential diagnostic sign, is one of the characteristics that sets MYH9-RD apart from other forms of the disease. Platelet macrocytosis in MYH9-RD is far more pronounced than in other inherited or acquired forms of thrombocytopenia, with giant platelets consistently present in blood smears. Routine automated cell counters frequently overestimate thrombocytopenia severity in MYH9-RD and fail to detect macrocytosis; thus, accurate platelet counts require microscopic examination or flow cytometry.^38^ Blood smear microscopy is also essential for identifying the hallmark macrocytic platelets.

Through the use of conventional blood smear staining, between 42% and 84% of cases with MYH9-RD have NMHCIIA inclusions. These inclusions, known as Döhle-like bodies, are faint and mildly basophilic, appearing in 15%–100% of neutrophil granulocytes. However, immunofluorescence staining for MYH9 protein reveals inclusions in all neutrophils of MYH9-RD patients, making immunofluorescence the gold standard diagnostic test.^39^ Research confirms nearly 100% sensitivity and specificity for MYH9-RD diagnosis using this technique.

Hearing loss is the most frequent late-onset symptom. Studies indicate that around 50% of MYH9-RD patients experience hearing loss by an average age of 33, with prevalence expected to rise over time. Hearing loss typically follows a progressive pattern of bilateral sensorineural impairment, initially affecting high and mid tones and eventually extending across all frequencies in severe cases.^40^ Early-onset forms, occurring in childhood or adolescence, often progress to severe or profound deafness.

About 25% of those diagnosed with MYH9-RD suffer from a renal condition, which often presents itself as progressive proteinuric nephropathy before the age of 35, with the average onset occurring at the age of 27. The progression of this type of nephropathy often occurs very quickly, leading to end-stage renal failure that necessitates either dialysis or transplantation.^41^ In spite of this, some cases manifest later in life and progress more slowly.

Presenile cataracts occur in approximately 20% of MYH9-RD patients, with a mean onset age of 37 years, although congenital cases have been noted. In most instances, cataracts are bilateral.^42^ About half of the people with MYH9-RD experience occasional or ongoing elevated liver enzymes, especially transaminases (ALT, AST) and gamma-glutamyl transferase. Fortunately, this increase is generally benign, with no reported cases leading to liver dysfunction.^43^ Unfortunately, to date, it is not possible to say whether individuals with MYH9 mutations are prone or resistant to malignancies.

### Pathogenesis of symptoms

The heavy chain of NMIIA is encoded by the MYH9 gene, critical for cytokinesis, cell adhesion, and cytoskeletal maintenance. In individuals with MYH9 mutations, while megakaryocytes appear structurally normal, defects occur in proplatelet branching, and impaired megakaryocyte migration may lead to abnormal platelet release into circulation.^44^ Bleeding tendencies in MYH9-related disorders are partly due to reduced platelet counts and impaired clot formation. Procoagulant platelets typically migrate to the thrombus surface with the help of clot contraction, but in the blood of MYH9-deficient mice, these platelets are unable to migrate and instead stay embedded in the thrombus.^45^

In kidney tissue, NMIIA is expressed in podocytes, mesangial cells, and tubular cells. Germline knock-in mutations in mice that mirror human MYH9 mutations have been linked to various disease manifestations, including albuminuria and glomerulosclerosis.^46^ However, findings are inconsistent regarding the spontaneous onset of glomerular disease in mice lacking MYH9, specifically in podocytes. Some studies suggest that these mice show increased sensitivity to stressors affecting podocytes, supporting human data indicating possible environmental or biological “triggers” that may promote chronic kidney disease progression. For example, mice with the MYH9 E1841K mutation (MYH9E1841 K/K/E1841 K) exhibit heightened sensitivity to certain renal stressors: mild albuminuria arises with high salt intake, severe albuminuria and focal segmental glomerulosclerosis develop under angiotensin II-induced hypertension, and reduced renal mass correlates strongly with decreased overall survival. Additionally, the activation of slit homolog 2 (SLIT2) and its receptor roundabout guidance receptor 2 (ROBO2) may stress podocytes by down-regulating MYH9 expression, increasing susceptibility to hypertension-induced podocyte detachment.^28^

MYH9-mutant and MYH9-deficient cultured podocytes display anomalies in the organization of their cytoskeleton, as well as increased motility and impaired mechanical performance. The conflicting results regarding the effects of podocyte-specific MYH9 deletion *in vivo* suggest the involvement of additional cell types in the disease progression. In addition to direct podocyte injury, glomerular pathogenesis may arise from disturbances in endothelial, mesangial, or tubular cells, as evidenced by human studies demonstrating focal, rather than widespread, podocyte foot process effacement. MYH9 localization has been established in mesangial, endothelial, and proximal, and distal tubular cells through multiple studies, indicating that dysfunction in these cells may also play a role in the pathogenesis of MYH9-related disorders.^47^

### MYH9 mutation spectrum

Over 80 distinct variations have been discovered in those diagnosed with MYH9-RD. A large number of these mutations are single-nucleotide changes, and they largely affect either the head domain or the coiled-coil region of the tail domain of the MYH9 gene. Arginine 702, which is found within the SH1 helix, which is an essential functional section, is the protein that undergoes mutations the most commonly within the head domain ^48^. Furthermore, a large number of mutations that are not located in the R702 cluster are in a particular hydrophobic region that is located at the interface between the SH3 motif and the upper 50 kDa subdomain. Some of the more complicated MYH9-RD phenotypes are associated with a number of the substitutions that occur at these sites (for example, to cysteine, histidine, or serine).^49^

Patients carrying R702 mutations often exhibit severe thrombocytopenia and are at high risk for developing aggressive nephropathy and/or progressive deafness. In approximately 20% of affected families, MYH9-RD is caused by splicing, nonsense, or frameshift mutations that affect intron 40 or exon 41, which leads to variable alterations of the non-helical tailpiece.^50^ In some rare cases, mutations arise from in-frame deletions or duplications, especially within exon 25, likely due to repetitive sequence patterns in this region.

**Figure S1:**
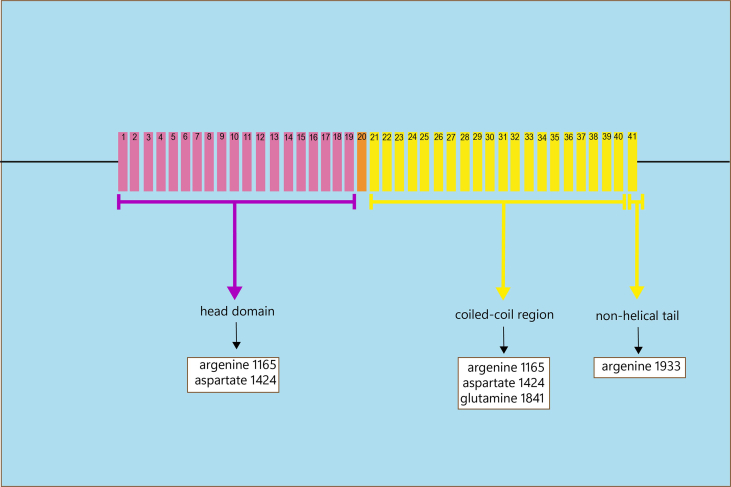
MYH9’s most common mutations that cause MYH9-RD.

Notably, around 70% of MYH9-RD cases stem from mutations at only six specific residues: serine 96 and arginine 702 in the head domain; arginine 1165, aspartate 1424, and glutamine 1841 within the coiled-coil region; and arginine 1933 in the non-helical tailpiece.^4^ Mutations at arginine 1165 and aspartate 1424 are also associated with increased risk for extra-hematological symptoms. Those diagnosed with the D1424H missense mutation, in particular, are frequently characterized by the development of proteinuria as well as deafness by their sixth decade and show heightened risk for cataract formation (Fig. S1).^51^

### Diagnosis and management

The initial suspicion for the identification of MYH9-RD relies on the outcomes of the hematological examination, as well as any extra-hematological manifestations that may be linked to the condition. The confirmation is accomplished by doing an immunofluorescence experiment on peripheral blood samples. This assay indicates the presence of myosin-9 inclusions that are typical for neutrophils.^33^ Proteinuria, which can occur with or without renal failure, is an indication that kidney involvement possesses some degree of significance. In order to diagnose hearing loss and cataracts, respectively, audiometric and ophthalmologic exams are necessary. In addition, around fifty percent of patients diagnosed with MYH9-RD also exhibit chronic or intermittent increases in liver-related enzyme levels.^52^

Molecular genetic methods may pinpoint the specific MYH9 mutation responsible, which aids in predicting the likely clinical progression. Differential diagnoses involve Bernard-Soulier as well as Alport syndrome, and immune thrombocytopenic purpura.^53^ Treatment is primarily symptomatic; however, establishing an etiological diagnosis is valuable for guiding therapy. This helps avoid inappropriate immunosuppressive treatments meant for immune thrombocytopenic purpura and enables the use of thrombopoietin receptor agonists, such as eltrombopag and romiplostim, to enhance platelet production. Eltrombopag has demonstrated efficacy in a phase 3 clinical trial.^54^

Renin-angiotensin system blockade has been proposed as a therapeutic approach for MYH9-RD-related nephropathy, with reports of reduced proteinuria and stable or slowly progressive renal function. However, responses vary, with some patients not responding to treatment. This variability may stem from the severity of certain genetic variants, which may be too advanced for nephroprotective strategies. In milder cases, renin-angiotensin system blockade may help protect against factors that exacerbate disease progression, such as renin-angiotensin system activation, which can worsen the condition by further reducing MYH9 expression.^55^

### ncRNAs affect MYH9 expression

In the past years, it was believed that ncRNAs have no function in the cell, but recent evidence highlighted the significance of ncRNAs in various diseases, particularly cancer, across humans and other species. Numerous studies have paid attention to the vital role of ncRNAs in the diagnosis and treatment of cancer, and ncRNAs related to *MYH9* have not been an exception to this order.^56^^,^^57^

ncRNAs consist of miRNAs, circular RNAs (circRNAs), and long non-coding RNAs (lncRNAs) play a crucial function in regulating the expression and activity of MYH9, a process critical to malignancy initiation, progression, and metastasis. They are distinguished by their length and structure. miRNAs are 21–23 nt, circRNAs are 100–10,000 nt, and lncRNAs are >200 nt in length.^58^

**Figure 2:**
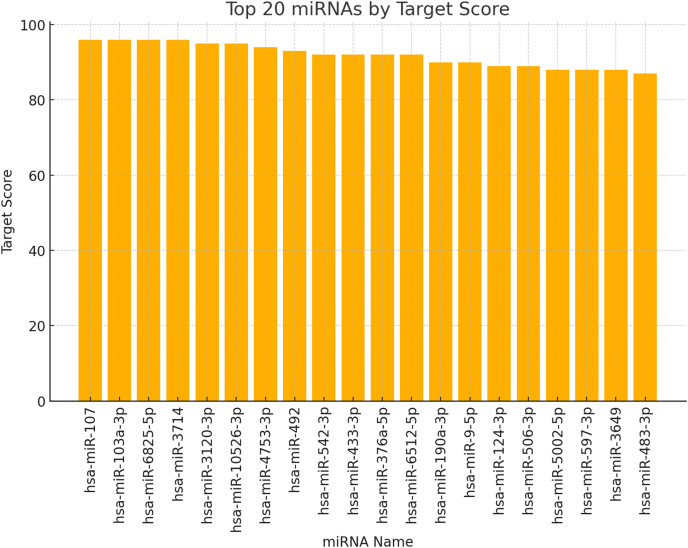
mirDB (https://mirdb.org/), an online database version 6.0, enables the prediction of miRNAs likely to target MYH9 based on Target Score, which is based on nucleotide pairing. Utilizing these predicted miRNAs can enhance our understanding of the regulatory mechanisms controlling MYH9 expression, providing valuable insights for further experimental validation.

miRNAs are small non-coding RNAs that have been found to play a pivotal function in the modulation of gene expression and are evolutionarily conserved. The first step for their generation consists of transcription carried out by RNA polymerases II and III, which leads to the formation of very long molecules, which are then shortened to yield functional miRNAs.^59^ miRNAs possess the ability to regulate essential biological processes, including cell differentiation, growth, and apoptosis (Fig. 2).^60^

As mentioned earlier, lncRNAs are characterized as non-coding transcripts exceeding 200 nucleotides in length, which have essential roles in multiple cell functionalities such as protein synthesis, cell lifespan, cell invasion in cancer, and other cancer-related tasks, which will be discussed in this paper.^61^

circRNAs are a type of non-coding RNA characterized by their covalently closed loop structures, typically derived from the exonic regions of protein-coding genes. These molecules are abundantly present across various cell types and are involved in numerous biological activities. In recent years, circRNAs have gained considerable interest due to their involvement in cancer development and their promise as biomarkers for diagnosis and potential therapeutic targets. Studies have indicated that circRNAs can influence the expression of MYH9 and contribute to cancer progression by interacting with miRNAs and through their capacity to encode proteins.^62^ The concept of competitive endogenous RNA (ceRNA), introduced by Salmena et al, suggests that RNAs containing miRNA response elements (MREs) can serve as molecular decoys. These decoys sequester miRNAs, thereby preventing them from binding to and repressing target mRNAs through their 3′ untranslated regions (3′UTRs).^63^^,^^64^

Gengtai and colleagues found that miR-647 inhibited the migration and invasion of gastric cancer cells by targeting the 3′UTR of SRF mRNA. This suppression of SRF allows it to interact with the CArG box within the MYH9 promoter, a mechanism linked to gastric cancer metastasis.^65^

In a separate study, Longyang et al reported that in human ovarian adenocarcinoma cell lines, elevated expression of miR-6089 reduced cancer cell proliferation, migration, and invasion by directly targeting MYH9, thereby disrupting the Wnt signaling pathway and preventing epithelial-to-mesenchymal transition.^66^

Hanwen et al worked on the role of lncRNAs in tumor progression of osteosarcoma, and they found that lncRNA MRPL23-AS1 was increased in osteosarcoma cell lines, and knocking down the lncRNA decreased cell invasion. It can decrease cell invasion by interacting with miR-30b, increasing *MYH9* expression, which leads to activation of the Wnt signaling pathway, so it promotes cell invasion in osteosarcoma patients.^67^

Another study on osteosarcoma showed that hsa_circ_0028173 (circATP2A2) can promote osteosarcoma by overexpression of MYH9 with the circATP2A2/miR-335-5p/MYH9 axis and also involves up-regulating glycolysis as well as cancer progression, so again MYH9 acts as an oncogene.^68^

In gastric cancer, hypoxia is essential for cell migration and invasion in gastric cancer cells. Xinhui et al discovered that by silencing circRNA SLAMF6 (circSLAMF6), which acts as a miR-204-5p sponge, MYH9 was a target for miR-204-5p. Inactivity of MYH9 suppressed migration and invasion in gastric cancer cells under hypoxia, which means MYH9 acts as an oncogene.^69^

Even in the colorectal cancer ceRNA network consisting of *MYH9* is available. Zili et al discovered that mir-214-3p suppressed the malignant behaviors of colorectal cancer by regulating the PLAGL2/MYH9 axis; thus, *MYH9* acts as an oncogene.^70^ Another study on colorectal cancer shows that up-regulation of miR-124 in cancerous cells can inhibit the expression of MYH9; the expression level of MYH9 in Caco2 and HCT116 cells decreases after transfection with miR-124, which suppresses cancer progression.^71^

In another study on colorectal cancer, Fan et al discovered that hsa_circ_0000395 was up-regulated in colorectal cancer, but the function is vague. Circ_0000395 sponges miR-432-5p to elevate MYH9 expression; it can facilitate colorectal cancer progression, and because of that, *MYH9* acts as an oncogene.^72^

MYH9 itself generates circRNAs, such as circMYH9, from its introns. Highly expressed in colorectal cancer, circMYH9 correlates with poor overall and recurrence-free survival, recruits hnRNPA2B1 in the nucleus, which leads to destabilization of pre-mRNA of p53, and its overexpression drives tumor growth by modulating serine/glycine metabolism and reactive oxygen species regulation in a p53-dependent manner.^73^

A study on gastric cancer demonstrated that lncRNA HULC modulates MYH9 expression by functioning as a sponge for miR-9-5p in gastric cancer cells. In the experiment, HULC exhibited up-regulation while miR-9-5p demonstrated down-regulation. Silencing of HULC and the subsequent reduction in MYH9 levels suggest that HULC silencing inhibits gastric cancer progression through the modulation of the miR-9-5p/MYH9 axis. As previously mentioned, MYH9 behaves as an oncogene in this study.^74^

A different study on gastric cancer revealed that circ-NRIP1 promotes glycolysis and cancer advancement through the miR-186-5p/MYH9 axis, whereas the circATP2A2/miR-335-5p/MYH9 axis facilitates both glycolysis and tumor progression.^75^

In pancreatic ductal adenocarcinoma, circSTX6 (hsa_circ_0007905) regulates the expression of MYH9 by sponging miR-449b-5p and by interaction with hypoxia-inducible factor 1-alpha (HIF1A) and Cullin2; it can up-regulate MYH9 expression, and MYH9 acts as an oncogene in pancreatic ductal adenocarcinoma.^76^

miR-124-3p can suppress the expression of MYH9 and other cytoskeletal genes, and it can perform its function unassisted. In neuroblastoma, decreased expression of miR-124-3p correlates with enhanced metastasis of cancer cells; consequently, MYH9 functions as an oncogene.^77^

circRNAs having protein-encoding potential represent a significant focus of research interest. Circ-EIF6 has been associated with unfavorable outcomes and adverse clinicopathological features in triple-negative breast cancer. It possesses a unique open reading frame (ORF) consisting of 675 nucleotides, along with an internal ribosome entry site (IRES) situated upstream of its ATG initiation codon. This structural arrangement enables the translation of a protein known as EIF6-224aa. The resulting protein contributes to cancer progression by stabilizing MYH9 and activating the Wnt/β-catenin signaling pathway.^78^

Thyroid cancer resistance to 131I radiation has been connected to the circ-NEK6/miR-370-3p/MYH9 axis, whereas non-small-cell lung malignancy resistance to cisplatin has been associated with the circ-PRMT5/miR-138-5p/MYH9 axis.^79^

lncRNA MAFG-AS1 is up-regulated in hepatocellular carcinoma cell line, MAFG-AS1 interacts with three subunits of NMIIA, including MYH9, MYL12B, and MYL6 and stabilizes them; thus, MYH9 acts as an oncogene.^80^ Furthermore, lincROR can stabilize MYH9 in prostate cancer by enhancing the β-catenin/hypoxia inducible factor 1 subunit alpha (HIF1α) pathway, perhaps serving as a critical factor in the resistance of prostate cancer cells to docetaxel.^81^

In summary, ncRNAs, including circRNAs, lncRNAs, and miRNAs, exert multifaceted regulatory effects on MYH9 through transcriptional, translational, and post-translational mechanisms (Table 2). Their precise regulation of MYH9 significantly impacts cancer progression, with their functions varying across cancer types and stages. Despite their promise as diagnostic and therapeutic targets, challenges remain in translating these findings into clinical applications, including the need for precise targeting of ncRNAs and minimizing off-target effects. Future research should adopt a broader perspective, exploring additional ncRNAs and cancer types to uncover their potential in cancer biology.

**Table 2:** The role of ncRNAs in various cancers by interacting with MYH9.

Tumor type	Mechanisms	MYH9 acts as	Reference
Gastric cancer	miR-647 lowers MYH9 expression and directly targets SRF mRNA to prevent gastric cancer invasion	Oncogene	65
	Inactivity of MYH9 can be suppressed in gastric cancer cell invasion by modulating circSLAMF6/miR-204-5p/MYH9	Oncogene	69
	circ-NRIP1 increases glycolysis and promotes disease progression by modulating the miR-186-5p/MYH9 axis	Oncogene	75
	lncRNA HULC controls MYH9 expression by functioning as a sponge for miR-9-5p in gastric cancer cells	Oncogene	74
Ovarian adenocarcinoma	To inactivate the Wnt/β-catenin signaling pathway and its downstream epithelial-to-mesenchymal transition (EMT), miR-6089 directly targeted MYH9	Oncogene	66
Osteosarcoma	lncRNA MRPL23-AS1 can interact with mir-30b and increase MYH9 expression and activation of wnt signaling pathway	Oncogene	67
	circATP2A2 can promote OS by modulating circATP2A2/miR-335-5p/MYH9 and it can up-regulates glycolysis	Oncogene	68
Colorectal cancer	mir-214-3p inhibits tumor growth and metastasis by targeting the PLAGL2-MYH9 axis	Oncogene	70
	MYH9 expression in Caco2 and HCT116 cells decreased following transfection with miR-124, which inhibits cancer progression	Oncogene	71
	Circ_0000395 can sponge miR-432-5p to elevate MYH9 expression, and it can ease colorectal cancer progression	Oncogene	72
	CircMYH9 is up-regulated in colorectal cancer. It can recruit hnRNPA2B1 in the nucleus, which leads to the destabilization of the pre-mRNA of p53	Oncogene	73
Pancreatic ductal adenocarcinoma	circSTX6 regulated the expression of MYH9 by sponging mir-449b-5p	Oncogene	76
Neuroblastoma	miR-124-3p can suppress the expression of MYH9 directly, which in neuroblastoma, mir-124-3p is down-regulated	Oncogene	77
Triple-negative breast cancer	Circ-EIF6 can hinder the degradation of MYH9 and promote the progression of triple-negative breast cancer via the Wnt/β-catenin signaling pathway	Oncogene	78
Hepatocellular carcinoma	MAFG-AS1 interacts with MYH9 and stabilizes it	Oncogene	80
Prostate cancer	By stimulating the β-catenin/HIF1α pathway, lincROR can stabilize MYH9 in prostate cancer	Oncogene	81

### MYH9 and cancer

#### The role of MYH9 in various malignancies

The MYH9 gene is frequently overexpressed in various cancers, including respiratory tumors such as lung cancer, reproductive system tumors like ovarian and prostate cancer, and digestive system malignancies such as hepatocellular carcinoma, colorectal cancer, and esophageal cancer.^82^ High MYH9 expression is also observed in hematologic malignancies, such as acute myeloid leukemia, and other cancers, facilitating tumor progression in many ways. Studies also suggested that up-regulated levels of MYH9 were associated with a poor outcome of gastric malignancy, and this could serve as a reliable biomarker for monitoring the progression and prognosis of cancer.^83^

MYH9 is highly expressed in colorectal cancer patients, with positive rates of 51.6% in colorectal adenocarcinoma tissues compared with 11.5% in paracancerous tissues. Its expression correlates significantly with age, clinical stage, lymph node metastasis, and distant metastasis in colorectal cancer.^84^ High levels of MYH9 expression in primary tumors have been linked to noticeably shorter survival times, according to Kaplan–Meier survival analysis. With median survival periods of 65.4 months (95% confidence interval/CI: 54.8–76.0) and 95.1 months (95% CI: 84.9–105.4), the five-year survival rates for patients with high and low MYH9 expression were 49% (31/63) and 86% (51/59), respectively. There was a statistically significant difference (*P* < 0.001).^85^ Additionally, multivariate analysis verified that MYH9 overexpression is a separate predictor of colorectal cancer outcome. Likely, in esophageal cancer, MYH9 was expressed in 100% of cancerous tissues compared with 50% in adjacent normal tissues. Multifactorial regression analysis revealed that MYH9 expression was significantly associated with lymph node metastasis (*P* = 0.015), tumor differentiation (*P* = 0.018), and advanced tumor staging (stages IIIB and IIIC, *P* = 0.007).^86^

In non-small cell lung cancer, MYH9 expression was observed in 38.3% of cases, but was absent in small cell lung cancer. Multivariate analysis revealed correlations between MYH9 expression and poor tumor differentiation, vascular invasion, and lymph node involvement.^87^ MYH9 was identified as an independent prognostic factor for non-small cell lung cancer (stages 1–3), with high MYH9 expression correlating with worse outcomes. Notably, patients with stage 1 non-small cell lung cancer who lacked MYH9 or vimentin expression had favorable prognoses following adjuvant chemotherapy.^30^

MYH9 is also highly expressed in osteosarcoma cells, with 75.38% positivity in immunohistochemical analyses. MYH9 expression is highly correlated with lung metastases and Enneking staging, but not with tumor size, patient age, or sex, according to clinical data.^88^ Overexpression of MYH9 promotes tumor invasion and metastasis, potentially through mechanisms related to the epithelial-to-mesenchymal transition, as demonstrated in breast cancer cells.^89^

It has been demonstrated that MYH9 overexpression activates the PI3K/AKT signaling pathway, resulting in increased levels of p-PI3K and p-AKT, which promote tumor growth. According to Zhao et al, nucleosome assembly protein 1-like 5 (NAP1L5) down-regulates MYH9 to suppress the PI3K/AKT/mTOR signaling pathway in hepatocellular carcinoma, exhibiting therapeutic benefits.^90^ Protein–protein interaction network analyses have suggested that Talin1 and MYH9 interact in acute myeloid leukemia, regulating key signaling pathways, including PI3K/AKT, to promote tumor cell proliferation and differentiation.^91^ Similarly, MYH9 significantly activates the PI3K/AKT/mTOR axis in esophageal cancer cells, enhancing tumor development and correlating with poor survival outcomes.

*In vivo* studies indicate that increased MYH9 expression enhances leukemia cell resistance to cytotoxic treatments, contributing to chemoresistance. In acute myeloid leukemia, high MYH9 expression is associated with specific miRNA markers. Research has revealed that the down-regulation of 18 miRNAs and the up-regulation of 3 miRNAs lead to elevated MYH9 levels.^92^ Among these, reduced levels of miR-188-5p are significantly correlated with poorer overall survival and event-free survival in patients with cytogenetically normal acute myeloid leukemia.^93^ Additionally, miR-16-1 serves as a prognostic marker in chronic myeloid leukemia, while miR-29c is a predictive biomarker for prognosis and treatment response in acute myeloid leukemia patients receiving cytarabine.^94^

MYH9 overexpression is particularly linked to the M4 subtype of acute myeloblastic leukemia, where patients often experience invasion of tissues such as the skin and bone marrow and exhibit resistance to chemotherapy. In the context of acute myeloid leukemia, MYH9 functions as a prognostic indicator, with elevated expression correlating with unfavorable outcomes. Clinical observations have demonstrated that acute myeloid leukemia patients with high MYH9 expression exhibit poorer prognoses.^95^ Multivariate analyses underscore this association, showing a statistically significant difference in overall survival between patients with high and normal MYH9 expression levels, with a hazard ratio (HR) of 1.69 (95% CI: 1.17–2.43, *P* = 0.005).

Clear cell renal cell carcinoma poses a significant clinical challenge because of its high degree of heterogeneity, which often results in unfavorable patient outcomes. Recent studies have highlighted the pivotal role of MYH9 in driving the development of clear cell renal cell carcinoma, primarily via the activation of the AKT signaling cascade. This MYH9/AKT signaling axis has been shown to influence how clear cell renal cell carcinoma cells respond to the targeted therapy drug sunitinib, implying that MYH9 could potentially be used as a predictive biomarker for assessing treatment effectiveness.^96^ In related research, Que and colleagues have discovered that in gliomas, HMGA1 promotes the MYH9-mediated ubiquitination of GSK-3β through the PI3K/Akt/c-Jun pathway, a mechanism that contributes to tumor aggressiveness and resistance to chemotherapy.^97^

MYH9’s influence extends to other pathways as well. Hou et al reported that inhibiting the β-catenin/MYH9 signaling pathway reduces ubiquitin protein ligase E3A (UBE3A) recruitment, which prevents UBE3A-mediated p53 degradation and deactivates the epithelial-to-mesenchymal transition pathway, thereby suppressing metastasis in nasopharyngeal carcinoma.^11^ Comparable mechanisms are observed in hepatocellular carcinoma, lung adenocarcinoma, diffuse large B-cell lymphoma, triple-negative breast cancer, and osteosarcoma.

**Figure 3:**
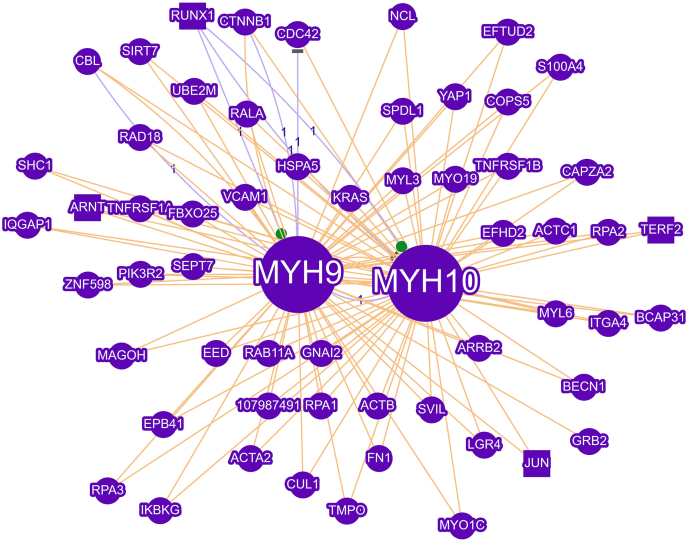
Interaction network of MYH9. MYH9 engages in multiple interactions with various genes associated with essential cellular functions.

MYH9 has also been identified as a crucial regulator of p53, a key tumor suppressor known for its role in limiting epithelial-to-mesenchymal transition. In colorectal cancer, elevated levels of circRNA circMYH9 disrupt the stability of p53 pre-mRNA by recruiting the nuclear protein hnRNPA2B1. This protein binds to N6-methyladenosine modifications within the 3′ untranslated region of the p53 transcript, resulting in decreased mRNA stability. Consequently, this reduction interferes with serine and glycine metabolic pathways and disturbs redox balance, ultimately enhancing tumor cell growth.^73^ Additionally, *in vivo* studies have shown that introducing circMYH9 through adeno-associated virus serotype 9 (AAV9) can suppress p53 activity and promote chemical-induced tumor formation in mice. In another study, Yang et al demonstrated that mucin 17 can inhibit the progression of gastric cancer by reducing inflammation through the MYH9-p53-RhoA feedback loop. Collectively, these studies underscore the significant role of MYH9 in tumor development, spread, and resistance to therapy, positioning it as a promising target for future cancer treatments.^98^ The MYH9 interaction network was analyzed using GenCLiP 3 (http://cismu.net/genclip3/analysis.php) (Fig. 3), and it was found that MYH9 has several interactions with various genes that are connected to fundamental cell activities.

### MYH9 as a novel target for cancer treatment

MYH9 has become known for its overexpression in a wide variety of tumor types and its involvement in malignancies growth. Recent studies indicate that MYH9 encodes NMII, and it serves as one important cytoskeletal protein exhibiting contractile forces required for the mobility of subcellular components and cell migration. This indeed opens up a promising opportunity to target NMII in treatment for cancer.^9^

### Small-molecule drugs targeting MYH9

Through a search that targeted NMIIA, blebbistatin, a tiny molecule that inhibits myosin, was discovered.^9^ It stabilizes myosin’s metastable state, which comes before the force-generating phase that is triggered by ATP hydrolysis, and functions as a non-competitive inhibitor. Blebbistatin’s pharmacological suppression of MYH9 in renal cell carcinoma suppresses the nuclear translocation of C-X-C chemokine receptor type 4 (CXCR4) and prevents the spreading of cancer cells.^99^ Blebbistatin further weakened the connection between MYH9, β-actin, and EGFR in different research on non-small cell lung cancer, which slowed the proliferation of cancer cells and hastened their demise.^100^ It possesses several limitations, such as fluorescence, low solubility in water, toxicity, and sensitivity to photodegradation, regardless of its extensive inhibitory effects on myosin isoforms.^101^

A bufadienolide isolated from toad venom, named CB, and the chemically synthesized CB have anti-cancer properties. To decrease cancer stemness, epithelial-to-mesenchymal transition, and cisplatin resistance in nasopharyngeal carcinoma, cinobufotalin raises MAP2K4 levels.^102^^,^^103^ MAP2K4 can suppress the PI3K/AKT/c-Jun pathway, which consequently suppresses MYH9 expression. Additionally, decreased MYH9 levels increase the cisplatin-induced chemosensitivity mediated by forkhead box O1 (FOXO1).^20^^,^^104^ Some other works have described a more elaborate way through which CB influences the regulation of MYH9. Studies have demonstrated that CB up-regulates ENKUR expression in lung adenocarcinoma and hepatocellular carcinoma by modulating the PI3K/AKT/c-Jun signaling pathway. The increased ENKUR can both directly modulate the function of MYH9 by the tail structural domain interaction, and also repress the mRNA level of MYH9 through the activation of the β-catenin/c-Jun pathway. In addition, changing the expression of MYH9 can regulate the binding to ubiquitin-specific protease 7 (USP7). It plays a part in the ubiquitination and degradation of cMyc, thereby playing an inhibitory role in epithelial–mesenchymal transition signaling and cancer progression.^103^ CB similarly suppresses MYH9 expression and stimulates ENKUR expression in nasopharyngeal cancer. But in this case, MYH9 suppresses the metastasis of nasopharyngeal cancer by reducing the recruitment of UBE3A, which prevents UBE3A from ubiquitinating and degrading p53.

The well-known chemotherapy drug called apatinib also has strong anti-angiogenic properties. Apatinib docking to thrombospondin 1 (THBS1) disrupts the ability of THBS1/MYH9 complex binding to glioma cells.^105^

Anti-hyperlipidemic drug bezafibrate can inhibit protein kinase C beta II (PKCβII) and PKCβII-triggered MYH9 phosphorylation in *in vivo* and *in vitro* assays; thus, breast cancer metastasis is prevented.^106^ J13, a small chemical that comes from *Albizia julibrissin*, increases mitochondrial fission in cancer cells by directly interacting with the MYH9-actin complex. Abnormalities in mitochondrial dynamics result from this overexpression. The growth, motility, and longevity of hepatocellular carcinoma cells are hindered by these changes, underscoring J13’s potential as an effective treatment for hepatocellular carcinoma.^107^ According to a different study, the small chemical methyl 2-(1H-indole-3-carbonyl)-thiazole-4-carboxylate (ITE) inhibits different glioma cell migratory pathways and stimulates endogenous aromatic hydrocarbon receptors (AHR).^108^ Astrocystin can promote anti-apoptosis and the spread of gastric cancer cells by focusing on cytosolic MYH9-induced catenin beta 1 (CTNNB1) transcription. This presents a new therapeutic strategy for stomach cancer metastases to the peritoneum. Furthermore, miRNAs target signaling pathways associated with MYH9.^9^

DT-13, known as saponin monomer 13 and derived from maitake, shows significant potential in inhibiting cancer metastasis across multiple cancer types.^109^ Studies indicate that DT-13, in conjunction with topotecan (TPT), facilitates EGFR degradation by inducing endocytosis through NMIIA, which subsequently inhibits hexokinase II (HKII) activity, suppressing aerobic glycolysis in BGC-823 cells. In low oxygen conditions, DT-13 further reduces metastasis in lung cancer by regulating NMIIA activity. In the tumor microenvironment, DT-13 inhibits the migration of cancer cells by down-regulating the c-raf/ERK1/2 pathway, leading to reduced NMIIA expression. Additionally, DT-13 influences the MYH9 in stromal cells, impeding breast cancer cell migration within the tumor microenvironment.^110^

Staurosporine suppresses MYH9 S1943 phosphorylation via reducing casein kinase II activity, which inhibits gastric cancer progression.^83^

The FDA has approved the medication disulfiram to treat alcoholism. After screening over 3185 chemicals, Robinson et al found that disulfiram (IC_50_ of 300 nM) was a strong growth inhibitor in triple-negative breast cancer cells. Disulfiram inhibits growth by directly targeting MYH9 and Ras GTPase-activating-like protein IQGAP1. The simultaneous use of doxorubicin and disulfiram improved the elimination of cancer stem cells.^105^

An aminated fullerene derivative called C70-EDA inhibits the NMIIA filament by binding to the C-terminal tail domain and altering MYH9’s subcellular distribution.^21^^,^^102^

Fullerenes that have been amide-functionalized have potent anti-cancer properties. A terminal amino group in the produced amphiphilic derivative of fullerene, TAPC-4, improves its capacity to bind MYH9, giving it a distinct molecular structure and amphiphilic characteristics.^111^

In acute and chronic myeloid leukemia, the natural alkaloid homoharringtonine (HHT) increases the expression of MYH9. Elevated MYH9 levels increase leukemia cell lines’ susceptibility to HHT-induced cell death.^112^

In Spitz tumors, oncogenic signaling driven by fusion proteins such as MYH9-NTRK3, ETV6-NTRK3, and MYO5A-NTRK3 can be effectively inhibited using DS-6051a, a compound that targets NTRK1/2/3 and ROS1 kinases.^113^

In the context of gastric cancer, MYH9 expression is down-regulated through the use of CCG-1423, a small-molecule inhibitor that blocks the Rho/SRF signaling axis, particularly in cancer cells with low levels of SRF. Notably, when CCG-1423 is administered alongside agomir-647 (a synthetic miRNA mimic), the combination exerts a potent, synergistic effect in suppressing the metastatic potential of gastric cancer cells.^65^

Rho GTPases function as molecular switches that are activated upon binding to GTP,^114^ subsequently triggering the activation of ROCK1 and ROCK2. These ROCK enzymes, classified as serine/threonine kinases, can become active in response to various stimuli, including mechanical tension, association with cellular membranes, and proteolytic processing. They control a number of functions, including immunological responses, cell motility, and differentiation. ROCK enzymes promote actomyosin contraction by phosphorylating myosin light chain 2 (MLC2), enhancing myosin II activity. They also activate LIM kinases to stabilize actin filaments. ROCK can activate LIM kinases, which prevent the disassembly of actin filaments. In cancer, the Rho–ROCK–myosin II pathway aids tumor growth, migration, treatment resistance, and immune response modulation. ROCK inhibitors can modify this pathway, making them potential treatments for cancer, fibrosis, glaucoma, and neurological disorders.^115^

Ripasudil is an approved medication used in the management of open-angle glaucoma and ocular hypertension. Beyond its ocular applications, it has been shown to enhance immune responses in patients with uveal melanoma by promoting the recruitment of CD8^+^ T cells, particularly when used alongside PD-1/PD-L1 immune checkpoint inhibitors.^116^ Netarsudil, another therapeutic agent, functions as a broad-spectrum ROCK inhibitor and is indicated for the treatment of elevated intraocular pressure in open-angle glaucoma. Pan-ROCK inhibitors like netarsudil have gained regulatory approval in both the United States and the European Union. Additionally, Belumosudil, a selective ROCK2 inhibitor, has received approval in the U.S. for treating chronic graft-versus-host disease in patients who are unresponsive to first-line therapies.^21^^,^^117^

Fasudil was the first ROCK inhibitor used in neurodegenerative disease. By preventing CD4^+^ T-cell differentiation and phagocytes, Fasudil suppressed neuroinflammation and reduced the release of inflammatory molecules, thereby reestablishing the equilibrium of immune cells *in vitro*. By lowering neuronal apoptosis brought on by ROCK overexpression in primary mouse hippocampus neurons, Fasudil also has a neuroprotective impact.^108^^,^^118^

Y27632 is an active inhibitor of ROCK that specifically targets PKA and PKC kinases, increasing doxorubicin effectiveness and tumor size reduction.^119^ It has been used in multiple myeloma, melanoma, and breast cancer. According to machine learning analysis, Fasudil and Y27632 both restore the effectiveness of BRAF inhibitors in resistant melanoma cells, and poorly differentiated tumors are more susceptible to ROCK inhibition.^120^ Despite failing in clinical trials, the ROCK/AKT inhibitor AT13148 has demonstrated promising outcomes in preclinical cancer models.^121^

Actomyosin contractility and metastasis depend on myotonic dystrophy kinase-related CDC42-binding kinase (MRCK). BDP5290 inhibits the migration of breast cancer cells by binding to the kinase domain of MRCK and blocking MLC phosphorylation.^122^

RKI-1447 and RKI-18 serve as potent Rho-kinase inhibitors that exhibit notable anti-tumor effects by inhibiting ROCK and MLC2 phosphorylation. This inhibition results in reduced formation of lamellipodia and filopodia in breast cancer cells while not impacting other kinases (Table 3).^123^

**Table 3:** MYH9 targeting drugs in cancer therapy: Summary of therapeutic agents that modulate MYH9 activity or expression in various malignancies, including their mechanisms of action and related tumor types.

Drugs	Mechanism and tumor	Reference
Blebbistatin	Non-competitive myosin-II inhibitors prevent cancer cells from becoming invasive in breast cancer	21,143
Cinobufotalin	Reduces expression of MYH9 in hepatocellular carcinoma and lung cancer and inhibits epithelial-to-mesenchymal transition and stemness in nasopharyngeal carcinoma.	10,104
Apatinib	Inhibits proliferation and migration, and phosphorylation of VEGFR2 in glioma cells	144
Disulfiram	Directly targets MYH9 and the Ras GTPase-activating-like protein IQGAP1 to prevent triple-negative breast cancer cell growth	105
C70-EDA	Prevents the formation of NMIIA filaments in A549 lung cancer cells	102
Fasudil	Rho-kinase inhibitor and calcium channel blocker	118
Amidated fullerene	Inhibition of cell migration and G0/G1 cell cycle arrest in various malignancies	14,102
Bezafibrate	Prevents breast cancer from metastasis	106
J13	Deactivating the molecular motors and weakening MYH9-actin connections to encourage the mitochondrial division process causes an imbalance in its dynamics and dramatically reduces the survival, proliferation, and migration of cancer cells	144
ITE	Inhibits different glioma cell migratory pathways and activates endogenous AHR	108
Astrocystin	Targeting the transcription of CTNNB1 cytosolically to prevent apoptosis and metastasis	9
Homoharringtonine	Up-regulates MYH9 expression in CML and acute myeloid leukemia cell lines	112
Staurosporine	Inhibits metastasis in gastric cancer	14,83
Y27632	ROCK1 and ROCK2 inhibitor	119
AT13148	Dual ROCK/AKT inhibitor	121
Fasudil	Rho-kinase inhibitor and calcium channel blocker	118
BDP5290	MRCK inhibitor	122
RKI-1447 and RKI-18	Rho kinase inhibitors	123
DS-6051a	Inhibits the fusions of MYO5A-NTRK3, ETV6-NTRK3, and MYH9-NTRK3 in spitz tumor	113
CCG-1423	Works effectively and synergistically with agomir-647 to prevent gastric cancer metastasis.	65

### Toxicity

Toxicity is the harmful effect of a substance when its dose exceeds the therapeutic limit, and it is considered cytotoxic if it damages cells or tissues. Blebbistatin demonstrates cytotoxicity (10–200 μM) in almost all cell types tested, and while it does not show cytotoxicity against U87 glioma cells (200 μM), it is cytotoxic against other cell lines.^124^ Apatinib has a tolerated maximum dose of 850 mg/day but exhibits adverse effects, including hypertension, fatigue, and diarrhea (causing treatment discontinuation in >55% of patients).^125^ Disulfiram can produce delayed toxicity (3–12 h later), harnessing a toxicity greater than 2–3 g in adults, and has a therapeutic dose of only 250 mg/day.^126^ Bezafibrate promotes apoptosis and causes liver and muscle toxicity (maximum tolerable concentrations of 300–1000 μM).^127^ ITE, C_70_-EDA, and RKI-1447 seem to show no toxicity in animal models.^128^^,^^129^ HHT produced dose-dependent toxicities (infections and skin rash) at the maximum tolerated dose.^130^ J13 was demonstrated to create powerful *in vitro* anti-cancer activity with little toxicity when tested on normoxic human primary hepatocytes.^107^ Y-27632 will produce necrosis and will cause inhibition of cellular proliferation at ≥200 μM, but is non-toxic at <100 μM.^131^ Studies show that CCG-1423 does not noticeably affect cortical neuron survival at doses up to 10 μM, even after 15 or 72 h.^132^ The optimal dose and toxicity profile of BDP5290 remain unclear and need further study,^133^ and STS is a highly potent and broadly cytotoxic drug, with its toxicity varying depending on the cell type.^134^

### MYH9 promotes tumor drug resistance

MYH9 has been linked to resistance in cancer treatments, such as levatinib in hepatocellular carcinoma and docetaxel in prostate cancer.^135^ High MYH9 levels can influence NOTCH signaling, contributing to levatinib resistance in hepatocellular carcinoma, while extracellular matrix density can reduce metformin efficacy.^136^ In prostate cancer, lincROR stabilizes MYH9, reinforcing the lincROR/MYH9/HIF1α pathway, which promotes docetaxel resistance. In lung adenocarcinoma, a MYH9-RETA fusion mutation has been associated with resistance to osimertinib.^81^^,^^137^ CB may reverse chemotherapeutic drug resistance associated with MYH9, according to many studies. This is achieved by suppressing PI3K/AKT signaling, which inhibits MYH9 transcription and down-regulates c-Jun, a negative transcription factor for ENKUR, increasing ENKUR expression.^10^ Although its effectiveness is restricted to certain cancer types, decreased MYH9 levels mitigate cisplatin resistance in lung adenocarcinoma by reducing USP7 recruitment, boosting c-Myc ubiquitination and degradation, lowering nuclear translocation, and deactivating epithelial-to-mesenchymal transition signaling.^9^

### MYH9-targeting immunotherapy

MYH9-targeting immunotherapies are a new method for cancer treatment, modifying the immune system to recognize and eliminate aberrant cells.^138^ Lung cancer is the most common cancer, making immunotherapy a crucial treatment for lung adenocarcinoma.^139^ Tang et al studied 200 patients with lung adenocarcinoma to investigate the possibility of fibrinogen-like protein 1 (FGL1) as a treatment option.^140^ Their research showed that FGL1 can affect the YY1-FGL1-MYH9 axis, a crucial immune-related cytokine, by modifying its production, affecting lung adenocarcinoma. Treatment options for Colorectal cancer patients with microsatellite-stable (MSS) tumors are severely limited by the low level of CD8 cytotoxic T lymphocytes. In both *in vivo* and *in vitro* studies, MAP7 domain-containing 2 (MAP7D2) knockdown significantly increased CD8 cytotoxic T lymphocyte infiltration, inhibiting tumor growth. Research shows MYH9 and MAP7D2 interact, protecting MAP7D2 from degradation and lowering high mobility group box 1 (HMGB1) secretion, inhibiting CD8 cytotoxic T lymphocyte infiltration in MSS colorectal cancer, potentially offering a new anti-tumor immunotherapy approach.^141^ Perforin applies tension to the less strong F-actin in tumor-regenerating cells through its interaction with non-muscle MYH9, according to another study. The interaction between tumor-regenerating cells and perforin enhances their stiffness, allowing perforin to pass through the cell membrane, facilitating the killing of tumor-regenerating cells and supporting tumor immunotherapy.^142^

## Conclusion

The MYH9 gene produces the myosin IIA non-muscle motor protein, a protein involved in signaling, motility, and cellular division. More recently, it has become related to tumorigenesis and resistance to therapies. The main purpose of this review is to present a critical overview regarding MYH9 acting as both a tumor suppressor and oncogene in a wide variety of cellular contexts. It also underlines the role of MYH9 in metastasis, treatment resistance, and connections with crucial signaling pathways such as Wnt/β-catenin and PI3K/AKT. MYH9-RD is characterized by thrombocytopenia and systemic consequences, raising intriguing considerations regarding the gene’s role in cancer propensity versus prevention.

New therapeutic strategies are emerging that aim to modulate MYH9-associated pathways. These include non-coding RNA-based therapeutics (miRNAs, circRNAs, and lncRNAs), small molecules such as blebbistatin and cinobufotalin, and immunotherapeutic approaches. However, translating these approaches into clinical trials remains a significant challenge, due to issues like tissue specificity, delivery efficiency, and off-target effects. Integration of nanoparticle-based delivery systems, CRISPR/Cas9 genome editing, and RNA-targeting technologies (*e.g.*, siRNA or antisense oligonucleotides) may help overcome some of these barriers and improve precision.

Through systematic analysis of ncRNA networks and pathway interactions, we demonstrate that MYH9 predominantly exerts oncogenic effects across malignancies by modulating invasion/metastasis, metabolic reprogramming (through glycolysis up-regulation), and canonical signaling cascades (Wnt/β-catenin and PI3K/AKT activation), with tissue-specific exceptions.

In pharmacokinetic terms, small-molecule inhibitors targeting MYH9 exhibit challenges like poor solubility, photodegradation, or fluorescence, but demonstrate therapeutic potential by modulating MYH9-related pathways in cancer. Meanwhile, ROCK inhibitors show varied pharmacokinetic profiles, with some achieving clinical approval for non-cancer indications, while others enhance chemosensitivity or immune responses in oncology but face limitations in specificity or trial outcomes.

Therefore, future studies should be directed toward elucidating context-dependent MYH9 functions in diverse types of cancers, developing effective targeted medicines, and using MYH9 as a biomarker for personalized treatment. Such filling of gaps in knowledge will provide an in-depth understanding of MYH9 in cancer biology. It will pave the way toward revolutionary treatment options that will eventually benefit patients suffering from MYH9-RD and MYH9-driven cancers.

## CRediT authorship contribution statement

**Shayan Emami:** Writing – review & editing, Writing – original draft, Methodology. **Amirreza Mazloomi:** Writing – review & editing, Writing – original draft, Visualization. **Fatemeh Ziadloo:** Writing – original draft, Visualization. **Shaghayegh Hosseinzadeh:** Writing – original draft, Methodology. **Hassan Saeedi:** Writing – original draft. **Azin Khoshghiafeh:** Writing – review & editing. **Mohammad Reza Ahmadifard:** Writing – review & editing, Writing – original draft, Supervision, Conceptualization.

## Conflict of interests

The authors declare that they have no known competing financial interests or personal relationships that could have appeared to influence the work reported in this paper.

## Abbreviations

MYH9: Myosin Heavy Chain 9
NMIIA: Non-Muscle Myosin IIA
MYH9-RD: MYH9-Related Diseases
ncRNA: Non-Coding RNA
miRNA: MicroRNA
lncRNA: Long Non-Coding RNA
circRNA: Circular RNA
EMT: Epithelial-Mesenchymal Transition
HCC: Hepatocellular Carcinoma
CRC: Colorectal Cancer
EC: Esophageal Cancer
AML: Acute Myeloid Leukemia
NSCLC: Non-Small Cell Lung Cancer
LUAD: Lung Adenocarcinoma
ccRCC: Clear Cell Renal Cell Carcinoma
TNBC: Triple-Negative Breast Cancer
ROS: Reactive oxygen species
OS: Osteosarcoma
PDAC: Pancreatic Ductal Adenocarcinoma
CTLs: Cytotoxic T Lymphocytes
MSS: Microsatellite Stable
CB: Cinobufotalin
DT-13: Saponin Monomer 13
HHT: Homoharringtonine
ROCK: Rho-Associated Coiled-Coil Containing Protein Kinase
MLC2: Myosin Light Chain 2
MRCK: Myotonic Dystrophy Kinase-Related CDC42-Binding Kinase
GVHD: Graft Versus Host Disease
AHR: aromatic hydrocarbon receptors
FGL1: Fibrinogen-Like Protein 1
TRCs: Tumor Regenerating Cells
